# Relevance of the Kappa Dynorphin System to Schizophrenia and Its Therapeutics

**DOI:** 10.20900/jpbs.20210015

**Published:** 2021-08-24

**Authors:** Anissa Abi-Dargham

**Affiliations:** Stony Brook University, 100 Nicolls rd, Stony Brook, 11794 NY, USA

**Keywords:** kappa opioid receptors, dynorphin, schizophrenia, antipsychotics

## Abstract

Multiple lines of evidence suggest a potential role for the kappa dynorphin system in schizophrenia and its therapeutics. Kappa stimulation acutely and chronically modulates dopamine in opposite ways, where acutely it decreases dopamine transmission and chronically it increases it and it can induce D2 sensitization. In addition, pharmacological evidence from studies using agonist and antagonists at KOR have indicated a therapeutic potential of KOR antagonists for psychosis. We present here a brief overview of the evidence supporting this viewpoint.

The opiate system includes the mu, delta and kappa opioid receptors (KOR). KOR is activated by the endogenous ligand dynorphin, a peptide neurotransmitter processed from its precursor prodynorphin [1]. KOR may play a role in schizophrenia based on clinical and preclinical evidence that we summarize briefly here. For more details we refer the reader to a prior extensive review [2].

KORs modulate both the dopamine neurons projecting to the striatum as well as those projecting to the cortex [3-5], providing a rationale for a postulated link to the dopamine imbalance in schizophrenia [6,7]. KORs are present on presynaptic axons of the mesolimbic and nigrostriatal pathways throughout the striatum [5,8,9]. KOR stimulation has differential effects on dopamine under acute vs chronic conditions. Acutely, KOR negatively regulate dopamine release from dopaminergic projection neurons [10] and may play an important role in maintaining dopamine homeostasis and synaptic plasticity [11,12]. Consistent with this mechanism, rodent studies in vivo have shown that systemic administration of an acute dose of selective KOR agonists reduces dopamine levels in mesolimbic and nigrostriatal pathways by acting on presynaptic KORs on dopaminergic neurons in striatal subdivisions [10,13-22]. Chronic KOR stimulation increases dopamine released in mesolimbic and nigrostriatal paths in response to stimulus or systemic administration of dopaminergic drugs. Chronic administration of the selective KOR agonist U69593 increases stimulus- and drug-evoked dopamine levels in both the mesolimbic [23-26] and nigrostriatal paths [26]. Similarly, chronic administration of Salvinorin A leads to increased drug-evoked dopamine levels in the nigrostriatal pathway [22]. Immediately following chronic administration, basal dopamine levels are unaltered and the ability of an acute systemic dose of U69593 to reduce dopamine levels is preserved, indicating that stimulated increases in dopamine are not due to KOR desensitization [5,23].

KORs form complexes with D2 autoreceptors of the nigrostriatal and mesolimbic pathways and postsynaptic D2 receptors on medium spiny neurons (MSNs) [5]. Stimulus- and drug-evoked DA release enhancement following chronic exposure to KOR agonists has been attributed to reduced presynaptic D2 autoreceptor function [23]. Supra-physiological levels of KOR signaling can accelerate and potentiate the locomotor sensitization to chronic administration of D2 agonists [5,27-30], via both pre and post-synaptic mechanisms. Timing of KOR activation and neural context of elevated dopamine levels play a critical role in the outcome of chronic KOR activation on sensitization [27], as KOR activation has the opposite effect on sensitization when administered sequentially with dopamine agonists rather than simultaneously [31-34]. The importance of neural context is further supported by acute studies of KOR on dopamine release showing that the effects of U50488 and Salvinorin A on drug- and stimulus-evoked dopamine response shift from inhibition to potentiation depending on the time of KOR agonist administration relative to stimulus [35,36].

Based on this evidence from preclinical studies and in light of the excess striatal dopamine function in schizophrenia underlying psychosis and its response to D2 blockade [37], and the evidence for supersensitized D2 receptors from imaging studies of comorbid schizophrenia and addiction [38], we propose that KORs may contribute to the positive symptoms of schizophrenia by interacting at least partly with the dopaminergic system to produce increased dopamine release and supersensitized D2 receptors and may be uniquely positioned to play a therapeutic role. This view is supported by studies of KOR agonists and antagonists that we summarize briefly below, and a meta-analysis that we have recently published [39]. A dopaminergic mediated effect of KOR agonists does not exclude potentially other non dopaminergic mechanisms that may lead to psychosis. In particular, the acute effects are not readily explainable by a dopaminergic mechanism, but could be related to other effects of KOR stimulation. KOR-expressing cells are present in cortical circuits, suggesting that DYNs released from cortical sources may act on KOR-containing cells within cortical microcircuits. KORs are present in presynaptic GABAergic terminals and inhibit GABA release from synaptosomes. DYN is also expressed in excitatory neurons in the PFC. This suggests a role for KOR in regulating the balance of inhibition excitation which may be involved in cortical circuitry dysfunction observed in schizophrenia. For an in depth review, see [40]. While speculative, this provides additional mechanisms for KOR involvement in the pathology of psychosis and cognitive dysfunction in schizophrenia and a potential for KOR based therapeutics.

KOR agonists are potent psychotomimetics in healthy people: cyclazocine, used to treat opioid dependence, and ketocyclazocine, both induced paranoid delusions and hallucinations [41-43]. Cyclazocine is a KOR agonist and a mu antagonist, and its psychotomimetic side effects were rapidly reversed by administration of the pan-opioid antagonist naloxone, indicating that psychotomimetic effects likely occurred through the kappa, rather than the mu opioid receptor [44,45]. Administration of the synthetic benzomorphan KOR agonist MR 2033/2034 as an analgesic resulted in the subjects experiencing psychotomimetic symptoms, including disturbances in the perception of space and time, visual hallucinations, racing thoughts, feelings of body distortion, and discomfort [46]. Similar to cyclazocine, these effects could also be blocked with naloxone. Trials with the selective KOR agonists enadoline, niravoline, and bremazocine as analgesics, and spiridoline for Parkinson’s disease, were discontinued due to psychosis in healthy volunteers [47-52]. More recently, salvinorin A, the active compound in the world’s most potent hallucinogenic plant *Salvia divinorum*, was found to be a selective KOR agonist with greater than 5000 times selectivity for kappa over mu and delta [53]. Numerous studies of salvinorin A in healthy volunteers have documented its psychotomimetic effects [54-56]. Most of these studies deal with acute effects. Chronic effects have not been studied to date.

Naloxone, naltrexone, and nalmefene, antagonists at the kappa, mu, and delta receptor, and buprenorphine, a dual KOR antagonist and mu partial agonist, have been tested in the treatment of SCZ with various results. We have recently reviewed these studies [2] and also performed a meta-analysis of all trials which showed a therapeutic signal for KOR antagonism [39]. Intravenous (IV) naloxone at dosages higher than 4 mg [44,57-63] produced significant improvement on the Brief Psychiatric Rating Scale (BPRS), while subcutaneous administration did not [64-68], possibly due to lower bioavailability [69]. A later trial using IV administration at appropriate dosage [70] failed due to a large placebo response. Naltrexone at 100 mg oral daily doses over a few weeks, a dose shown to produce 87% KOR occupancy [71], was generally successful for treating the positive and negative symptoms of schizophrenia [72,73]. Another study of daily naltrexone at 100 mg dose augmentation in 60 patients stabilized on risperidone for 12 weeks [74] found significant improvement of both positive and negative symptoms compared to placebo. However, other smaller, or shorter duration studies using naltrexone at dosages greater than 100 mg did not demonstrate efficacy [75-79]. Two studies showed positive results with 0.2 mg of sublingual buprenorphine, a mu opioid receptor partial agonist and KOR antagonist [80,81]. Authors concluded that the antipsychotic effects of buprenorphine were most likely mediated through the KOR. Similarly, nalmefene in a double-blind placebo-controlled crossover trial in 10 patients with schizophrenia on antipsychotics [82] showed some improvement. Overall two studies showed a therapeutic signal [74,82] when KOR antagonists were added to D2 antagonists. These findings suggest an additive effect of KOR antagonism with D2 antagonism, which should be formally tested and confirmed in future studies.

In addition to this data in psychosis, a recent study showed a therapeutic signal of KOR antagonists in the treatment of anhedonia. This was documented in a Phase II-A, 8-week, double-blind, parallel-group, placebo-controlled, fixed-dose study of JNJ-67953964 10 mg vs placebo in patients meeting DSM-5 mood or anxiety disorder diagnostic criteria who also had anhedonia (Snaith Hamilton Pleasure Scale Score ≥20) [83]. This provides additional rationale for testing KOR antagonists in schizophrenia in light of the reward disturbances present in this illness.

In summary, KOR have modulatory effects on the dopaminergic system, and potentially also on cortical inhibitory and excitatory balance. KOR antagonists have a potential for therapeutic effects on both positive and negative symptoms of schizophrenia. Considerable heterogeneity in the pharmacological data however suggests the need for a better understanding of the neurobiology and the development of biomarkers to stratify potential candidates for treatment with these agents. In vivo molecular imaging of the KOR may offer some insights into this heterogeneity. In particular, [^18^F]LY2459989 is an antagonist radiotracer recently developed and validated in human and nonhuman primates [84,85]. An antagonist radiotracer is essential to avoid any potential side effects from stimulating the KOR. This tracer exhibits favorable pharmacokinetic and in vivo binding characteristics, including an appropriate rate of metabolism, a reliably measurable free fraction in arterial plasma, high brain uptake, fast and reversible tissue kinetics, and specific and selective binding to the KOR. Studies using this tracer may help to classify patients with pathology of the KOR system in order to target specific interventions to increase the likelihood of effectiveness.
